# Comparative Analysis of the Complete Chloroplast Genome of Four Known *Ziziphus* Species

**DOI:** 10.3390/genes8120340

**Published:** 2017-11-24

**Authors:** Jian Huang, Ruihong Chen, Xingang Li

**Affiliations:** 1 Forest Key Laboratory of Shaanxi Province, College of Forestry, Northwest A & F University, Yangling 712100, China; 2 Shaanxi Province Key Laboratory of Jujube, College of Life Science, Yan’an University, Yan’an 716000, China; chenruihong328@163.com

**Keywords:** *Ziziphus*, chloroplast genome, phylogenetic analysis, microsatellite markers, *Ziziphus spina-christi*

## Abstract

*Ziziphus* Mill. (Rhamnaceae) is comprised of about 170 species that are mainly distributed in tropical to subtropical regions, with few in the temperate zone. Several *Ziziphus* fruit tree species are important energy, nutrient, and medicinal resources for human populations, particularly for those living in rural regions. To date, limited genomic information is available for this genus. Here, we assembled the complete chloroplast genomes of four best known *Ziziphus* species, i.e., *Ziziphus jujuba*, *Ziziphus acidojujuba*, *Ziziphus mauritiana*, and *Ziziphus spina-christi*, based on the Illumina Paired-end sequencing method. The chloroplast genomes of the four *Ziziphus* species are all very similar to one another, and exhibit structural, gene content, and order characteristics that are similar to other flowering plants. The entire chloroplast genome encodes 113 predicted unique genes (85 protein-coding genes, 8 rRNA, and 37 tRNA), 17 of which are duplicated in the inverted repeat regions. Rich single sequence repeats loci (217) were detected in *Z. jujuba* and 106 SSR loci, composed of A/T, displayed polymorphism across the four species by comparative genomic analysis. We found only four genes under positive selection between *Z. jujuba* and *Z. acidojujuba*, and two genes for Z. *mauritiana* vs. *Z. spina-christi*, respectively, while half of the 78 protein-coding genes experienced positive selection between the two groups. Phylogenetic analyses revealed that *Ziziphus* (Rhamnaceae) was sister to Elaeagnaceae, and the four species of *Ziziphus* were clustered into two groups (*Z. jujuba* and *Z. acidojujuba*, *Z. mauritiana* and *Z. spina-christi*). Our results provide genomic resources for intrageneric classifications of *Ziziphus*, and valuable genetic markers for investigating the population genetics and biogeography of closely related *Ziziphus* species.

## 1. Introduction

*Ziziphus* (Mill.: Rhamnaceae) is comprised of about 170 species widely distributed in tropical and subtropical regions, including a few in the temperate zone [1,2]. Most species of *Ziziphus* are regionally distributed in developing countries and have received little attention. A few species of *Ziziphus*, particularly those growing in Asia, Africa, and South America, produce edible and multi-nutritional fruits, supplementing human diets and to farmers income [1]. For example, Chinese jujube (*Ziziphus jujuba*) has become the most widely planted *Ziziphus* fruit tree in the temperate zone. *Z. jujuba* and its progenitor, *Ziziphus acidojujuba*, native to the North China, were introduced to central Asia and Europe, including Iran, Israel, and Romania through the “Silk Road” trade thousands of years ago, and more recently to North America [2,3]. *Ziziphus mauritiana*, known as India jujube, is native to a wide tropical region from Vietnam, India to Sahel, and domesticated in India. India jujube has been introduced to Taiwan, South China, and North Africa [4,5]. Other well-known species, *Ziziphus spina-christi* (L.) Willd. known as Christ’s thorn, is common from Sudan and Ethiopia in Africa, to Lebanon, Syria, and Iran in the Middle East. It produces small fruits, and is widely used in folk medicine and as a source of food and energy for rural populations [6,7]. Although the whole genome of *Z. jujuba* has been sequenced [8,9], there is still little genomic information about other species in *Ziziphus* for intrageneric classifications.

Chloroplasts are semi-autonomous organelles in plant cells, and contain a circular chromosome ranging from 120 to 165 kb in length, which possess several chloroplast-specific genes involved in photosynthesis, carbon fixation, starch biosynthesis, etc. Chloroplast genome is uniparentally inherited, and its gene content, placing order, and base composition is exceptionally conserved between plant species. Therefore, the chloroplast genome has been considered as a desirable “barcoding” for phylogenetic studies in higher taxa. On the other hand, non-coding regions in chloroplast genome showed higher polymorphism than coding regions, and are frequently used to evaluate population genetic structure at intrageneric and intraspecies levels. Benefiting from significant advances in next generation sequencing technologies, increasing numbers of chloroplast genomes have been assembled. Sequencing and phylogenetic analysis on the complete chloroplast genome is a highly efficient and low-cost way for improving intrageneric classifications and population analyses.

Intrageneric classifications of *Ziziphus* is a thorny dilemma, due to the different views and limited genomic information relative to the large number of species in *Ziziphus*. Islam and Simmons (2006) [1] constructed the phylogenetic relationships of 19 species of *Ziziphus* based on nuclear (internal transcribed spacers (ITS) and 26S rDNA) and plastid (*trnL-F*) genes. In this study, we assembled the chloroplast genomes of the four best known *Ziziphus* species (*Z. jujuba*, *Z. acidojujuba*, *Z. mauritiana*, and *Z. spina-christi*) based on the Illumina Hiseq Platform, and detected chloroplast simple sequence repeat (SSR) loci for resolving population structure. In addition, we performed phylogenetic analyses on the four species and reconstructed the phylogenetic position of *Ziziphus* within the order Rosales. The chloroplast genomes and SSR markers described in this study will not only provide genomic resources for intrageneric classifications of *Ziziphus*, but also allow us to explore the population genetic structure and domestication events.

## 2. Materials and Methods

### 2.1. Samples

Young leaves of the four species were harvested with the intent of total DNA extraction. *Z. jujuba* “Junzao” and *Z. acidojujuba* “Qingjiansuanzao” were grown at the jujube experimental station of Northwest A & F University (37.1° N, 110.1° E), Qingjian, Shaanxi Province, China. *Z. mauritiana* “No.1 Gaolang” was grown at the Institute of Tropical Eco-Agriculture of the Agriculture Academy of Yunnan Province (25.7° N, 101.9° E), Yuanmou, Yunnan Province, China. *Z. spina-christi* fruits were collected from Khartoum, Sudan in August 2016. Sampling was performed according the Sudanese law about collection and exportation of biological material for scientific studies, and under the regulation of cooperation and exchange between Northwest A & F University in China and the University of Nyala in Sudan. *Z. spina-christi* seeds were sown and grown in the greenhouse of Northwest A & F University with controlled temperature conditions (28 ± 5 °C/20 ± 5 °C day/night). Young leaves were collected from one seedling (voucher number: SCNW-01) for DNA extraction.

### 2.2. Genome Sequencing

We performed high quality total DNA extraction using the DNeasy Plant miniKit (QIAGEN, Valencia, CA, USA). The purity of extracted DNA was evaluated based on the ratio of absorbance at 260 nm/280 nm and 260 nm/230 nm using a NanoDrop 1000 spectrophotometer (Thermo Scientific, Wilmington, DE, USA) and its concentration (28 ng/μL) was measured using a Qubit fluorometer (Invitrogen, Carlsbad, CA, USA). The degradation of DNA was checked by gel electrophoresis. 

We constructed a paired-end sequencing library with insert sizes of 180 bp. For constructing libraries, 5 mg of DNA were sheared to fragments of 180–800 bp using a Covaris ultrasonic disruptor (Covaris, Woburn, MA, USA). Fragments were end-repaired, A-tailed, and ligated to Illumina paired-end adapters following standard protocols. Then, the DNA libraries were sequenced on Illumina HiSeq platforms, and the generated sequence reads were deposited in GenBank under accession numbers: SRX1506571 (*Z. jujuba*), SRX1526053 (*Z. acidojujuba*), SRX3373470 (*Z. mauritiana*) and SRX3380018 (*Z. spina-christi*).

### 2.3. Chloroplast Genome Assembly

The generated reads were first subjected to quality control and filtering using Illumina CASAVA 1.8.2. We first discarded the paired reads when either one read contains an adapter and a read length cut-off of 50%. The filtered reads with over 20% of their nucleotides having a *Q* score < 20 (probability of sequencing error > 0.01). 

A combination of de novo and reference-guided assembly was used to perform jujube chloroplast genome assembly. Filtered reads were imported into Geneious R 10.1.3 (Biomatters Ltd., Auckland, New Zealand) and assembled according to [10,11]. Briefly, reads were firstly de novo assembled into contigs, and those contigs belonging to chloroplast genome were separated by alignment to the reference chloroplast genome of *Pyrus pyrifolia* (NC_015996). The aligned contigs were ordered according to their position in the reference genome. Thirdly, the assembled draft genomes were mapped again with obtained clean reads. 

To verify the accuracy of sequence assembly, regions with ambiguous read mapping (i.e., conflicted reads mapped to the same genomic region) and low coverage were verified by PCR amplification and Sanger sequencing. We designed 81 primer sets (Supplementary Material Table S2) based on assembled sequence to amplify 77 non-coding sites and four junctions between single-copy region and inverted repeats. The chloroplast genomes of the other three *Ziziphus* species were assembled with reference to the assembled chloroplast genome of *Z. jujuba*, using the reference-guided assemblage strategy as described in [10]. Finally, the assembled four-chloroplast sequences were deposited in GenBank. The de novo assembly were performed on a computer equipped with two CPUs (Intel XeonE5-2600v2) and eight Kinston SIMMs (16G DDR3/1600 RECC), and the reference-guided assembly were performed on a MacBook Pro (CPU: 2.2 GHz Intel Core i7, memory: 16GB 1600MHz).

### 2.4. Genome Annotation

The assembled genome sequences were preliminarily annotated in Geneious. The exact start-and-stop codons of protein-coding genes and exon boundaries were verified by BLASTX against the GenBank/DDBJ/EMBL database. The tRNA genes were further confirmed through online tRNAscan-SE web servers [12]. The gene map of annotated jujube chloroplast genome was drawn by OGDraw v 1.2 online [13].

### 2.5. Identification of Repeat Sequences and Simple Sequence Repeats

Repeat sequences in chloroplast genomes were detected by REPuter online [14]. We included the three types: direct, reverse, and palindromic. The constraints were set to (i) minimum repeat size of 30 bp, and (ii) 90% or greater sequence identity, based on a Hamming distance of 3. Gap size between palindromic repeats was restricted to a maximal length of 3 kb. Overlapping repeats were merged into one repeat motif whenever possible. If multiple types of repeat were detected in the same region, only one repeat type was designated. The tandem repeat was determined to be prior to the dispersed repeat if one repeat motif could be identified as both tandem and dispersed. To avoid redundancy, one of the two inverted repeat (IR) regions was removed prior to identification of repeat motifs. 

The SSRs were searched using WebSat software, with the following repeat threshold settings: 10 repeats for mono-nucleotide, 5 for di-, 4 for tri-nucleotide, 3 repeats for tetra- and penta-nucleotide SSRs [15].

### 2.6. Genome-Wide Sequence Variations and Gene Selective Pressure Analysis among Four Ziziphus Chloroplast Genomes

To investigate divergence in chloroplast genomes, the identity across whole chloroplast genomes were visualized using the mVISTA viewer among the four species, based on full alignments with annotations [16]. Variation, including single nucleotide polymorphisms (SNP) and indels, were detected using the “find variation” function in Geneious. The percentage of the variable sites was calculated for each region (coding regions and non-coding regions).

The evolutionary rate of the chloroplast genome sequence was quantified based on nonsynonymous (dN) and synonymous (dS) substitutions and their ratios (ω = dN/dS). dN and dS were computed according to the LWL85, LPB93, and LWLm methods [17,18,19] using PAML 4.7.1 [20], with the F3 × 4 codon-based substitution model. Only codons shared among all chloroplast genomes were compared. dN, dS, and ω were calculated for 78 protein-coding genes, respectively. Six pairwise alignments for each gene region were calculated.

### 2.7. Phylogenetic Analysis

Seventy-eight common protein-coding genes were extracted from the four *Ziziphus* species and 27 close taxa chloroplast genomes, including one species belonging to Vitaceae, 16 to Rosaceae, one to Ulmaceae, five to Moraceae, and one to Rhamnaceae for phylogenetic analysis (Supplementary Material Table S3). Amino acid sequences were aligned in MUSCLE [21], manually adjusted, and then constrained back to the nucleotide alignment. The phylogenetic analyses were performed on the nucleotide substitution matrix using three methods: maximum parsimony (MP), maximum likelihood (ML), and Bayesian inference (BI). ML analyses were performed in MEGA 6.0.6 [22], with a 10,000× bootstrap test. Although the good fitness model was inconstant among different genes, the General Time Reversible (GTR) incorporated with the Gamma distribution and invariant sites (G+I) with the lowest BIC scores (Bayesian Information Criterion) from the 24 models was considered to describe the substitution pattern for the combined protein-coding gene sequences (Supplementary Material Table S3). The gap/missing data in the aligned matrix data were deleted. The heuristic method was selected for searching the ML tree by topological rearrangements of an initial tree. MP was performed in PAUP 4.0 [23] with 1000 random addition replicates and tree bisection reconnection (TBR) branch swapping with “Multrees” option. We used a subtree-pruning-and-regrafting (SPR) algorithm to search for the optimal tree under the maximum likelihood (ML) and maximum parsimony (MP) criterion. BI was analyzed in MrBayes 3.1.1 [24] with a “GTR+G+I” model, two runs with four Markov chains with heating values of 0.01 (2 × 10^7^ generations in total), sampled every 100 generations until convergence. The first 25% of burn-in trees were discarded, and remaining trees were used to construct a majority-rule consensus tree, while the frequency of inferred relationships was used to estimate posterior probabilities (PP). 

## 3. Results

### 3.1. Genome Assembly and Validation

In total, 240,608,000 raw reads (125 bp) generated from paired-end sequencing were used for *Z. jujuba* assembly. By screening these paired-end reads through alignment with reference chloroplast genomes, 12,672,000 reads were mapped to the reference genome. After de novo and reference-guided assembly, the chloroplast genome of *Z. jujuba* with 161,215 bp was obtained. Four junction regions between IRs and SSC/LSC and intragenic regions were confirmed by PCR amplification and Sanger sequencing. The amplified sequences amounted to approximately 47,841 bp (29.6%). In addition, we compared these sequences directly to the assembled genomes and observed few nucleotide mismatches or indels. This result validated the accuracy of our genome sequencing and assembly. Therefore, the other three species were assembled by 117×, 225.9×, and 149.4× reads coverage for *Z. acidojujuba*, *Z. mauritiana*, and *Z. spina-christi* with reference to the chloroplast genome of *Z. jujuba* using the reference-guided strategy, respectively (Supplementary Material Table S1). The size of chloroplast genomes is over 160 Kb, *Z. acidojujuba* (161,211 bp) is close to *Z. jujuba*, while *Z. mauritiana* (161,543 bp) and *Z. spina-christi* (161,615 bp) showed similar size. These genome sequences were deposited into GenBank under accession numbers (KX266829, KX266830, KY628304, KY628405).

### 3.2. Genome Structure, Gene Content, and Features

**Figure 1:**
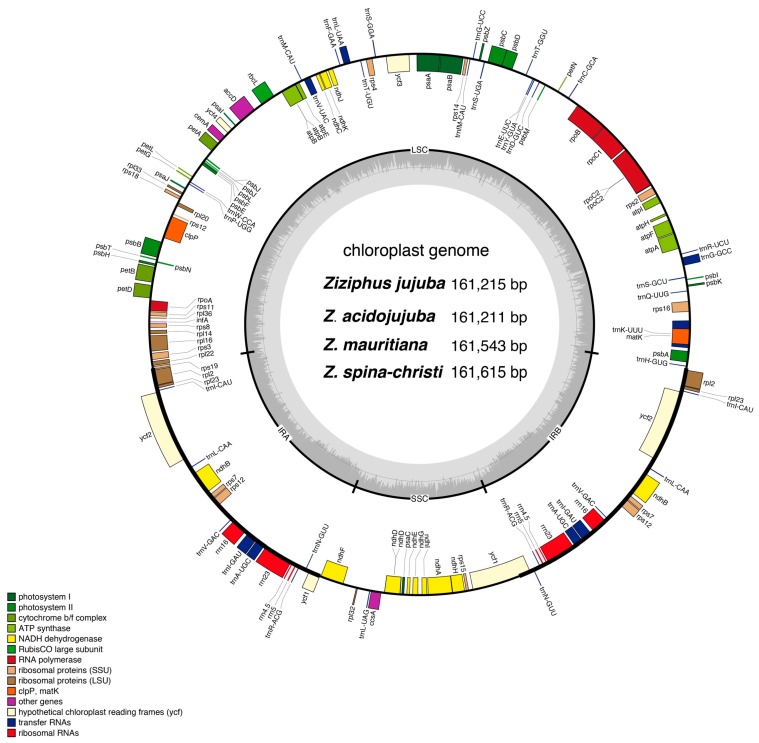
Chloroplast genome assembly, size and features of four *Ziziphus* species. Genes lying outside of the outer layer circle are transcribed in the counter clockwise direction, whereas genes inside are transcribed in the clockwise direction. The colored bars indicate different functional groups. The darker gray area in the inner circle denotes GC content while the lighter gray corresponds to AT content of the genome. LSC: large single copy, SSC: small-single-copy, IR: inverted repeat.

The chloroplast genome of the four *Ziziphus* species exhibited a circular DNA molecule with a typical quadripartite structure. It consisted of two-inverted repeat regions (IRa and IRb) separated by large (LSC) and small (SSC) single-copy regions, respectively (Figure 1). After gene annotation, we found the four chloroplast genomes displayed synteny of gene order that harbored 113 unique genes including 85 protein-coding genes, 8 rRNA, and 37 tRNA, 17 of which were duplicated in the IR regions, 12 in SSC region and 84 in LSC region. Among these unique genes, 16 included single intron, two (*ycf3*, and *clpP*) have two introns (Table 1). One of trans-spliced *rps12* exons is in the LSC region, and two reside in the IR regions separated by an intron. Coding regions accounted for 58.8% of the whole genome, and 41.2% are inter-genic spacers and introns. All four chloroplast genomes were AT-rich (~63.2%), and the ratio of A and T in *Z. jujuba* varied slightly among non-coding regions, protein-coding regions, tRNA, and rRNA, in which A+T contents were 66.60%, 61.03%, 57.94%, and 52.19%, respectively.

### 3.3. IR Expansion and Contraction

**Figure 2:**
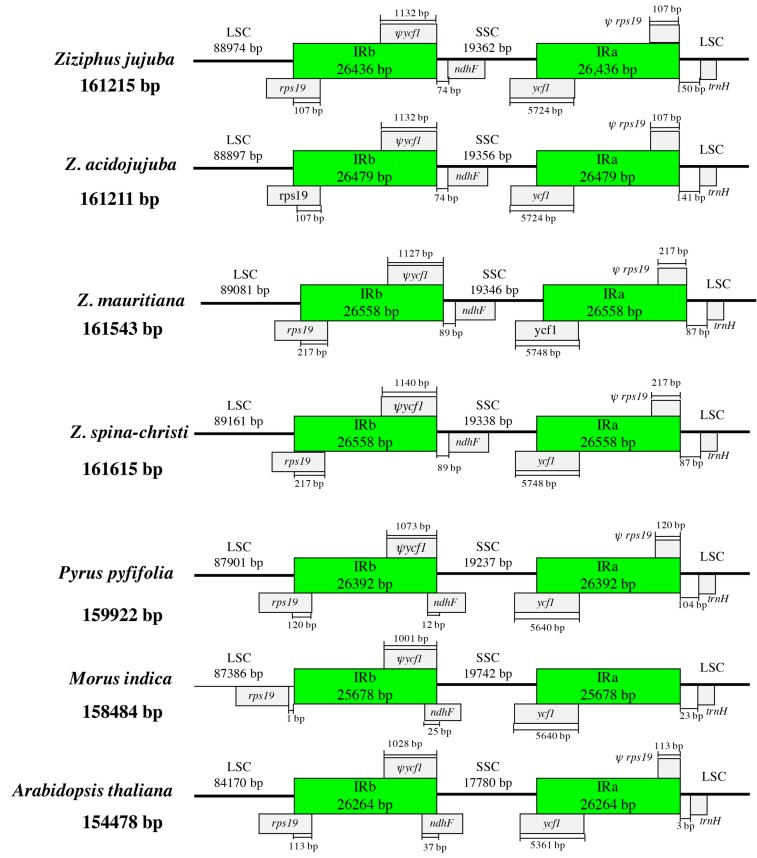
Comparisons of LSC, SSC, and IR border regions among the four *Ziziphus* chloroplast genomes.

Chloroplast genome structure and the junction positions between LSC/IRs and SSC/IRs were well conserved between the four *Ziziphus* species. LSC, SSC, and IRs sections of the four chloroplast genomes were larger than those corresponding sections of reference species (*Pyrus pyfifolia*, *Morus indica*, and *Arabidopsis thaliana*), which contributed to the larger chloroplast genome of *Ziziphus* species (Figure 2). For *Z. jujuba*, the IRb/SSC border (position 115,410) was in the 3′ region of the *ycf1*, and created a *ycf1* pseudogene in IRb with a length of 1132 bp. The IRb/LSC border (position 88,974) was located within the coding region of *rps19*. Correspondingly, a 3′-truncated *rps19* pseudogene with a length of 107 bp was created by IRa/LSC border (position 161,215). In addition, a 150 bp length of the intergenic region existed between IRa/LSC border and *trnH-GUG*. The chloroplast genome of the four species of *Ziziphus* showed similar structure. In particular, the IR region of *Z. mauritiana* and *Z. spina-christi* have the same size (Figure 2). We selected two phylogenetically close species (*P. pyfifolia* and *M. indica*) and the model species (*A. thaliana*) as references to compare the chloroplast genome structure. Only the LSC/IRb junction of *M. indica* located in *rps19*, as well as the 3′ truncated *rps19*, disappeared in the border of IRa/LSC. IRb contracted from *ndhF* in *Ziziphus* while it expanded into *ndhF* in other three species. 

### 3.4. Repetitive Sequences

A total of 27 and 28 repeats were detected in *Z. jujuba* and *Z. acidojujuba*, respectively, and 26 loci were common to both species. On the other hand, 27 and 25 repeats were identified in *Z. mauritiana* and *Z. spina-christi*, respectively, and 24 were common to both species (Supplementary Material Table S4). However, 14 loci of repeats were shared in *Z. jujuba/Z. acidojujuba* and *Z. mauritiana/Z. spina-christi*. This pattern displayed the distribution of repeats that is rather conserved in the two groups. The size of repeat was in the range of 30–40 bp. Forward repeats are the most common type and accounted for 66.7%, 67.9%, 65.4%, and 66.7% of total repeats in *Z. jujuba*, *Z. acidojujuba*, *Z. mauritiana*, and *Z. spina-christi*, respectively. Almost all repeats are distributed in non-coding regions, only two repeats were found in two genes (*psaB* and *ycf2*) in *Z. jujuba* and *Z. acidojujuba*, and an additional two repeats are present in *accD* of *Z. mauritiana* and *Z. spina-christi*.

### 3.5. Single Sequence Repeat Polymorphism

**Figure 3:**
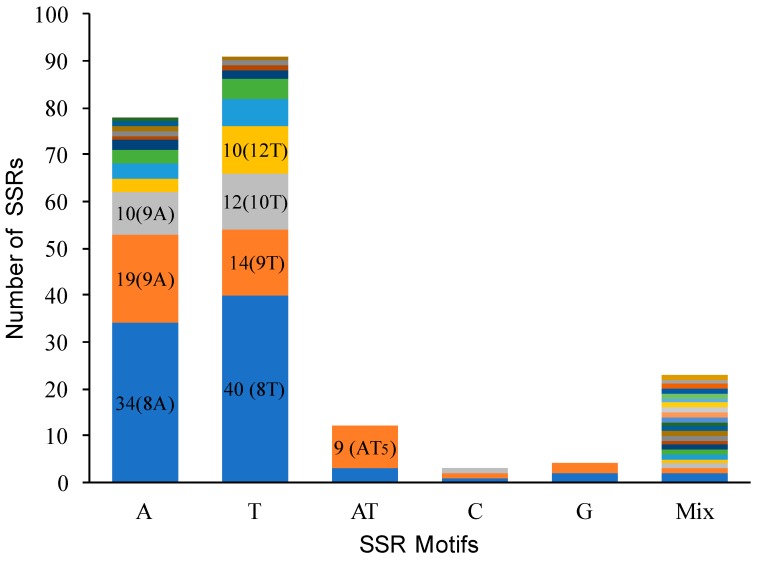
Frequency of different simple sequence repeat (SSR) types in the chloroplast genome of *Z. jujuba*.

We identified 217 SSR loci across the four species, and found almost all SSR were located at the same locus in the four *Ziziphus* species (Supplementary Material Table S5). Four sections of the chloroplast genome (LSC, SSC, IRs) contained 163 SSR loci, 34 SSR loci, and 20 (10 × 2) SSR loci, respectively. Among those SSR loci, 159 (73.95%) distributed in 75 of 133 (56.39%) intergeneric regions, 30 (13.95%) in the coding regions, and 26 (12.09%) in introns. We found 39 intergeneric regions contained greater than one SSR locus and 16 intergeneric regions contained approximately four to six SSR loci. For example, *ndhF-rpl32* and *trnS-GCU*-*trnG-GCC* contained six SSR loci. In addition, *clpP* intron contained 11 SSR loci, and *ycf1* exon contained 11 SSR loci. In general, T/A were the major motif types of SSR loci, 91, 77, and 12 SSRs were mononucleotide stretches of T, A, and AT in *Z. jujuba*, respectively (Figure 3). Among these, 106 SSR loci showed polymorphisms that were mainly contributed from the variation (74 loci) between two groups (*Z. mauritiana*/*Z. spina-christi* vs. *Z. jujuba*/*Z. acidojujuba*). In addition, 10 SSR loci only occurred in *Z. mauritiana* and *Z. spina-christi*, 21 loci specially presented in *Z. jujuba* and *Z. acidojujuba*, one locus, only, occurred in *Z. jujuba*. 

### 3.6. Global Sequence Variation across Chloroplast Genomes

**Figure 4:**
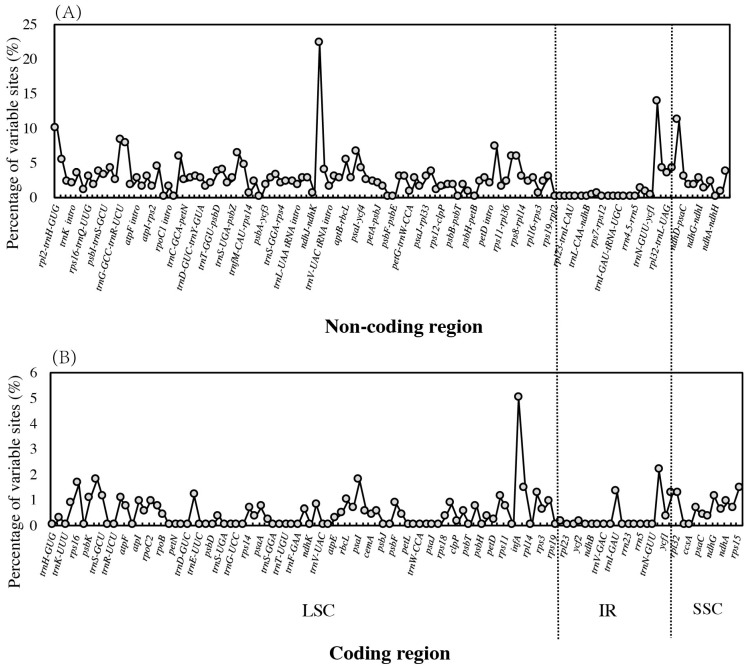
Nucleotide variability (%) values compared among four *Ziziphus* species. (**A**) non-coding regions; (**B**) coding regions.

Based on the global identity of the chloroplast genome, *Z. acidojujuba* vs. *Z. jujuba* (99.6%), *Z. mauritiana* vs. *Z. spina-christi* (99.9%) are more similar while the identities were 97.3–97.5% between the two group members. mVISTA plotting also demonstrated this pattern among the four species. The marked differences were mainly found in LSC and SSC region between the two groups, while the IRs are more conserved than single-copy regions (Supplementary Material Figure S1). In total, 1940 variable sites, including 442 indels, 610 transitions, 792 transversions, and 96 substitutions, were detected across the four chloroplast genomes. Only 322 variants were detected between *Z. mauritiana* and *Z. spina-christi*, while 1306 variants were found between *Z. jujuba* and *Z. acidojujuba*. There were 11 indels with more than 30 bp, among which the largest indels reached 170 bp in the intergenic region between *accD* and *psaI*. In addition, we found three other large indels with the length of 107 bp, 71 bp, and 21 bp in the same region. In contrast, coding regions (0–22.22% variability, mean value: 2.60%) are more conserved than non-coding regions (0–5.00%, mean value: 0.45%; Figure 4). The highest variable coding regions was *infA* (5.00%), followed by *ycf1* (2.15%), *psbI* (1.80%), *psaI* (1.75%), and *rps16* (1.63%). The most divergent non-coding regions were *ndhK–ndhC* (22.22%), *ycf1–ndhF* (13.70%), *ccsA–ndhD* (11.11%), and *rpl2–trnH–GUG* (10.00%). No variation was observed in 52 coding regions and 20 non-coding regions between the four species. (Supplementary Material Table S6). 

### 3.7. Evolutionary Rates

We found very low values dN and dS from *Z. jujuba* vs. *Z. acidojujuba* and *Z. mauritiana* vs. *Z. spina-christi*, and only four genes (*ycf1*, *rpoA*, *ccsA*, and *atpI*) and two genes (*ycf1* and *ndhK*) have *ω* values. Particularly, *ycf1* and *ccsA* have very high values 2.6 and 1.5, though dN and dS were fewer than 0.01 (Supplementary Material Table S7). In contrast, 39 protein-coding genes had *ω* values from 152 pairwise comparisons, which were generated between *Z. jujuba*/*Z. acidojujuba* and *Z. mauritiana*/*Z. spina-christi*. Those genes covered all the functional types, such as photosynthetic system, ribosomal proteins, NADH dehydrogenase, *ycf* etc. Among which, the ω value of two genes (*rpl16* and *rps12*) exceeded 1.0 in four species. Eight genes had ω values in the range from 0.5 to 1.0, i.e., *ycf3*, *rpl22*, *clp*, *ycf1*, *accD*, *rpl20*, *ycf4*, and *psbK*.

### 3.8. Phylogenetic Analysis Based on the Protein-Coding Genes

**Figure 5:**
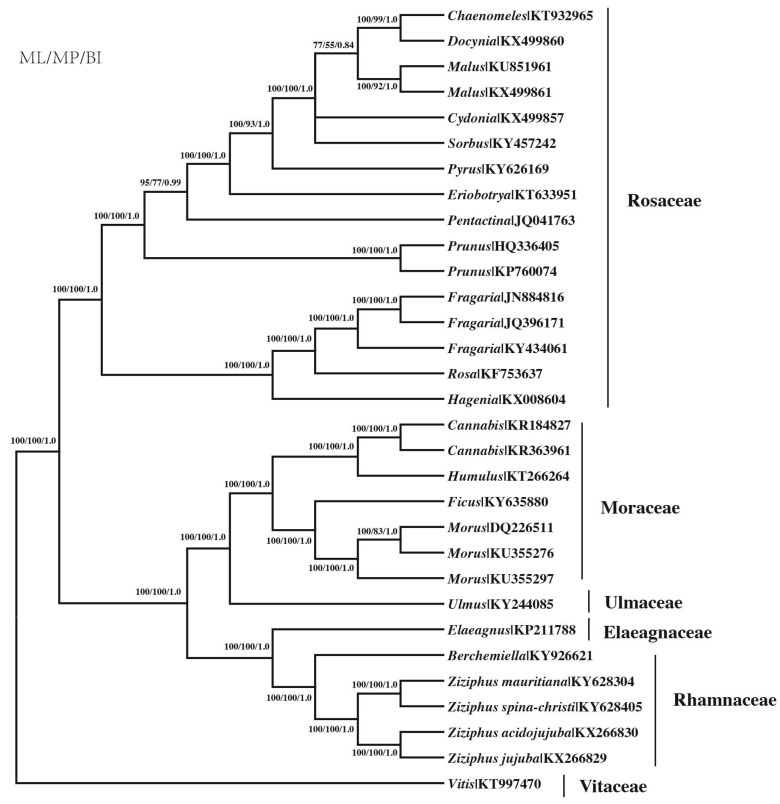
Phylogenetic tree reconstruction of the 31 species inferred from maximum likelihood (ML), maximum parsimony (MP) and Bayesian inference (BI), based on 78 protein-coding genes using a non-partitioning scheme. Numbers above the lines represent: ML/MP bootstrap values/BI posterior probability.

All three phylogenetic methods resulted in one fully resolved and congruent topology of the 31 taxa tree (Figure 5). ML analysis under the GTR+G+I model resulted in a tree with Maximum Likelihood value (lnL) = −171,890.05, BIC (344,765.8) and Akaike Information Criterion (AIC) 343,918.11. All clades at the family level were strongly supported by all methods with BI and ML support generally higher than for MP. Twenty-one of the 29 nodes were completely supported by 100% bootstrap and one PP support values, and seven additional nodes had >55% BS in MP tree. The tree was generally divided to two large groups, Rosaceae and four other families. Four species of *Ziziphus* initially clustered with a *Berchemiella* species belonging to Rhamnaceae, and then grouped with Elaeagnaceae, Ulmaceae and Moraceae in order. *Ziziphus* species were divided into two groups: *Z. jujuba* and *Z. acidojujuba*, *Z. mauritiana*, and *Z. spina-christi*.

## 4. Discussion

We assembled the chloroplast genomes of four known species of *Ziziphus* (*Z. jujuba*, *Z. acidojujuba*, *Z. mauritiana*, and *Z. spina-christi*). The *Ziziphus* chloroplast genome contained 113 unique genes. This gene number and content of *Ziziphus* chloroplast genome are in the range of most of the angiosperm plastid genomes [25]. Based on sequence similarity, genome structure, gene content, and phylogenetic analysis, the four species can be clearly separated into two groups: e.g., *Z. jujuba* and *Z. acidojujuba*, *Z. mauritiana* and *Z. spina-christi*. Islam and Simmons (2006) [1] found that *Z. jujuba* and *Z. acidojujuba*, and *Z. mauritiana* and *Z. spina-christi*, clustered together based on three genes, i.e., nuclear (ITS and 26S rDNA) and plastid (*trnL-F*) genes. This classification was obviously related with geographical distance of their native ranges. Both *Z. jujuba* and *Z. acidojujuba* were deciduous species, traditionally co-distributed in the mid and lower reaches of the Yellow River. Additionally, *Z. jujuba* was domesticated from *Z. acidojujuba*, which would contribute to their close phylogenetic relationship [9]. On the other hand, *Z. mauritiana* and *Z. spina-christi* are evergreen species, and native to tropical and subtropical regions, and overlap in their geographical distribution. *Z. mauritiana* is widely distributed, from South Asia to North Africa, and *Z. spina-christi* occurs from north Africa to Iran. Like the relationship between *Z. jujuba* and *Z. acidojujuba*, they showed high similarity in their chloroplast genome sequences. This pattern also proved the close relationship between *Z. mauritiana* and *Z. spina-christi*. It was additionally reflected by the success of interspecific crossing among *Z. mauritiana*, *Z. spina-christi*, and *Z. jujuba*. Asatryan and Tel-Zur (2013) [26] found a higher total percent of fruit set in *Z. mauritiana* × *Z. spina-christi* crosses, while only a few fruits were obtained from the *Z. jujuba* × *Z. spina-christi*, and *Z. mauritiana* × *Z. jujuba* crosses.

Chloroplast SSR markers are potentially useful genetic resources to investigate population genetics and biogeography of closely related taxa. In this study, comparative analysis of the four chloroplast genomes revealed 217 SSR loci, almost all of which occurred in the same loci. There are often fewer than 100 SSR loci as reported in many other species, such as 67 in *P. pyrifolia*, and 63–71 SSRs in *Cardiocrinum* [27,28]. This difference was primarily ascribed to different statistic methods. Many non-coding regions contained more than one SSR locus, each of which was treated as one locus, resulting in more loci in this study than some other species from previous reports. In addition, most loci (106 SSR loci) displayed polymorphisms across the four species that were mainly attributable to the difference (74 loci) between two groups (*Z. mauritiana*/*Z. spina-christi* vs. *Z. jujuba*/*Z. acidojujuba*). This pattern demonstrated the strength of the SSR marker for resolving genetic relationships between the closely related taxa. In a previous study, we demonstrated the population structure of *Z. jujuba* and *Z. acidojujuba* based on six chloroplast SSR markers and demonstrated the domestication pathway of *Z. jujuba* [29]. Therefore, those polymorphic SSR loci provide valuable genetic resource for investigating the population genetics and biogeography of closely related *Ziziphus* species.

Although the chloroplast genomes of higher plants are highly conserved, sequence variation in the non-coding region was generally higher than the coding region and each locus (gene/intergeneric region) showed different variations (Figure 4). Highly divergent regions were often regarded as “hotspots” and used as genetic markers for population genetic or phylogenetic studies. In this study, we found *ycf1* was the largest gene in *Ziziphus* chloroplast genome, and harbored the highest divergence (2.15%). The *ycf1* gene has also been shown to be phylogenetically useful among *Orchidaceae* [30], *Solanum* [31], and *Magnoliidae* [32]. Wang et al. [33] also suggested that *ycf1* deserved consideration in phylogenetic analyses in angiosperms by comparing 78 protein-coding genes with *ycf1* alone, especially for reconstructing the phylogeny of *Rosaceae* with higher node supports. Thus, *ycf1* might be used as a valuable gene marker for *Ziziphus*. On the other hand, we found these highly divergent regions varied across taxa. For example, we found *rps19* had the highest proportion of variability in genic regions of *Cardiocrinum* [28] and *Camellia* [34]. This pattern indicated their independent/distinct evolutionary patterns for different genes in different clades.

Our analysis revealed few genes were under positive pressure in two groups, i.e., *Z. jujuba* vs. *Z. acidojujuba*, and *Z. mauritiana* vs. *Z. spina-christi*, respectively. Previous studies have clarified that *Z. jujuba* was domesticated from *Z. acidojujuba*, accompanied with a transition from bushes to trees and enlarged fruit sizes [9,29]. In addition, *Z. mauritiana* and *Z. spina-christi* have overlapping geographical distributions and similar biological features. In contrast, half of those protein-coding genes were under positive selection between the two groups, i.e., *Z. jujuba*/*Z.acidojujuba* and *Z. mauritiana*/*Z. spina-christi*, which might be ascribed to the shift of habitat from temperate zone to tropic. For instance, *clp* was under positive selection, with ω of 0.8. Zheng et al. (2002) [35] revealed *clp* proteins are primarily constitutive proteins of chloroplast stroma, and were involved in plant acclimation to different physiological conditions. In addition, *accD* have a ω of 0.7. Kode et al. [36] provided a direct evidence that the *accD* gene could affect plant fitness and is required for leaf development. Thus, the leaves of *Z. mauritiana*/*Z. spina-christi* are obviously enlarged compared to *Z. jujuba/Z. acidojujuba* might be linked to the selection effect on this gene. As expected, *ycf1* gene have a high ω value from 0.68 to 2.63 between the four species. Because *ycf1* had been classified as the most divergent coding sequence in plastome of vascular plants, and therefore, contributed to fast evolution [37]. Taken together, our results revealed that genes were undergone selection might be involved in the adaptation to the shift of environment condition during the evolution between *Z. jujuba*/*Z.acidojujuba* and *Z. mauritiana*/*Z. spina-christi*, and provide valuable genetic resource for investigating the population genetics and biogeography of closely related *Ziziphus* species.

## Figures and Tables

**Table 1:** List of genes encoded by four species of *Ziziphus* chloroplast genome.

Category	Group	Genes
Self-replication	Large subunit of ribosome (LSU)	rpl2(×2)*, *rpl14*, rpl16*, *rpl20*, *rpl22*, *rpl23(×2)*, *rpl32*, *rpl33*, *rpl36*
	Small subunit of ribosome (SSU)	*rps2*, *rps3*, *rps4*, *rps7(×2)*, *rps8*, *rps11*, rps12*(×2, *part)*, *rps14*, *rps15*, rps16*, *rps18*, *rps19*
	*DNA dependent RNA polymerase*	*rpoA*, *rpoB*, rpoC1*, *rpoC2*
	*Ribosomal RNA*	*rrn4.5(×2)*, *rrn5(×2)*, *rrn16(×2)*, *rrn23(×2)*
	Transfer RNAs (tRNA)	trnA-UGC(×2)*, *trnC-GCA*, *trnD-GUC*, *trnE-UUC*, *trnF-GAA*, trnG-GCC*, *trnG-UCC*, *trnH-GUG*, *trnI-CAU(×2)*, trnI-GAU(×2)*, trnK-UUU*, *trnL-CAA(×2)*, trnL-UAA*, *trnL-UAG*, *trnM-CAU*, *trnfM-CAU*, *trnN-GUU(×2)*, *trnP-UGG*, *trnQ-UUG*, *trnR-ACG(×2)*, *trnR-UCU*, *trnS-GCU*, *trnS-UGA*, *trnS-GGA*, *trnT-UGU*, *trnT-GGU*, *trnV-GAC(×2)*, trnV-UAC*, *trnW-CCA*, *trnY-GUA*
Photosynthesis	Photosystem I	*psaA*, *psaB*, *psaC*, *psaI*, *psaJ*, ycf3**, *ycf4*
	Photosystem II	*psbA*, *psbB*, *psbC*, *psbD*, *psbE*, *psbF*, *psbH*, *psbI*, *psbJ*, *psbK*, *psbL*, *psbM*, *psbN*, *psbZ*, *psbT*
	NADH dehydrogenase	ndhA*, ndhB(×2)*, *ndhC*, *ndhD*, *ndhE*, *ndhF*, *ndhG*, *ndhH*, *ndhI*, *ndhJ*, *ndhK*
	Cytochrome b/f complex	*petA*, petB*, petD*, *petG*, *petL*, *petN*
	Subunits of ATP synthase	*atpA*, *atpB*, *atpE*, atpF*, *atpH*, *atpI*,
	Large subunit of rubisco	*rbcL*
Other genes	Translational initiation factor	*infA*
	ATP-dependent protease subunit p gene	clpP**
	Maturase	*matK*
	Envelop membrane protein	*cemA*
Unknown function	Subunit of acetyl-CoA-carboxylase	*accD*
	c-type cytochrome synthesis gene	*ccsA*
	Hypothetical chloroplast reading frames	*ycf1(×2)*, *ycf2(×2)*

(×2) following gene indicate 2 copies; One and two asterisks indicate one- and two-intron containing genes, respectively.

## Supplementary Materials

The following are available online at www.mdpi.com/2073-4425/8/12/340/s1. Table S1 Chloroplast genome assembly, size and features, Table S2 PCR primer sets for chloroplast genome sequence validation, Table S3 Species and gene lists used for phylogenetic analyses, Table S4 Repeats (≥30 bp) identified in four *Ziziphus* species, Table S5 SSR position in *Ziziphus* chloroplast genome, Table S6 Percentage of variation sites in each region, Table S7 Nonsynonymous substitution (dN), Synonymous substitution (dS), and dN/dS (ω) values for individual genes or gene groups (ω(dN, dS), Figure S1 Sequence identity plots among the four Ziziphus chloroplast genomes viewed by mVISTA, with *Z. acidojujuba* as the reference. Annotated genes are displayed along the top. The vertical scale represents the percent identity between 50 and 100%. Genome regions are color coded as exon, intron, and conserved non-coding sequences.
