# Supplementary figures and images for: Comparative Analysis of the Complete Chloroplast Genome of Four Known Ziziphus Species
Source: Genes (Basel). 2017 Nov 24;8(12):340. doi: 10.3390/genes8120340 (PMC5748658; doi:10.3390/genes8120340)

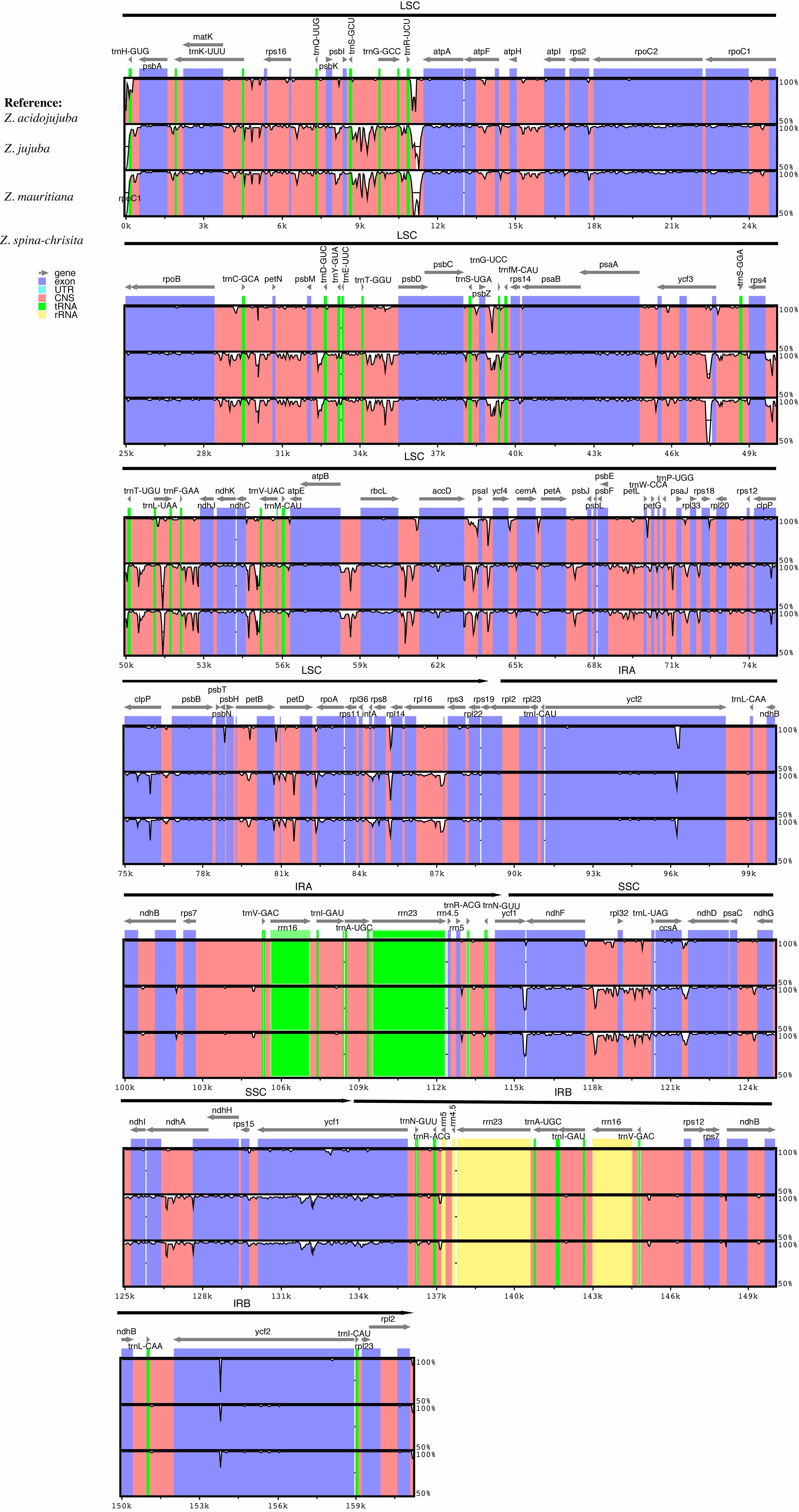

Supplement: Supplementary file 1 [file genes-08-00340-s001.zip › Supplementary Figure 1 gpvista.jpg]
